# Challenges in Diagnosis and Management of Second Trimester Omental Pregnancy in Limited Resource Settings: Case Report

**DOI:** 10.24248/eahrj.v6i1.673

**Published:** 2022-07

**Authors:** Beata Nyangoma Mushema, Beno Steven Nkwama, George Alcard Rweyemamu, Isaac Hamis Makanda, Monica Chiduo

**Affiliations:** a Department of Obstetrics and Gynaecology, Hubert Kairuki Memorial University, Dar es Salaam, Tanzania; b Morogoro Regional Referral Hospital, Morogoro, Tanzania

## Abstract

**Background:**

Abdominal pregnancies are a rare occurrence and are associated with high maternal and perinatal mortality rates compared to intra-uterine and other ectopic pregnancies. Localization of sites of abdominal pregnancies and determining the gestational age at the time of diagnosis play a fundamental role in guiding the treatment approach and minimizing potential complications. However, the vague presentation coupled with low accuracy of ultrasound detection often leads to misdiagnosis of abdominal pregnancy, and hence delaying initiation of appropriate management. We present a case of a second trimester abdominal pregnancy detected following failure of induction for an initial diagnosis of missed abortion, and the ensuing outcome, rarely reported in limited-resource settings.

**Case presentation:**

A 19 year old unbooked woman, gravida 2 para 1 at 17^th^ week gestation age by ultrasound presented with loss of foetal movement for one week. Based on clinical assessment and referral ultrasound findings, she was initially diagnosed with missed abortion and planned for induction. Failure of induction prompted a repeat ultrasound which revealed a non-viable abdominal pregnancy. Laparotomy was done, localisation of the pregnancy at the omentum was observed and a dead foetus was extracted with the placenta left in-situ. A complication of surgical wound dehiscence with infection developed post-operatively and was managed with secondary sutures. The patient recovered and was discharged in a stable condition.

**Conclusion:**

This case demonstrates that the diagnosis of abdominal pregnancy remains a challenge especially in settings where skilled human resources for health are few and equipment and supplies for effective and timely treatment are limited. The case sheds some light on the broader challenges in maternal and perinatal health in developing countries. Accurate pre-operative diagnosis requires a high index of suspicion, especially due to the variability of its presentation. This case emphasises the important of quality antenatal care and the need for clinicians to conduct comprehensive assessments of patients and receive training on obstetric ultrasound skills.

## BACKGROUND

Abdominal pregnancies occur when there is implantation of the blastocyst in the peritoneal cavity, external to the uterine cavity and fallopian tubes. These pregnancies are rare, accounting for approximately 1% of all ectopic pregnancies,^1^ and are associated with a high maternal mortality and perinatal mortality rate compared to intra-uterine and other ectopic pregnancies.^2^ Estimated incidence of abdominal pregnancies has been reported to be 1 in 10,000 live births,^3^ with the omentum, pouch of Douglas, pelvic side wall, spleen, bowel and liver included as potential implantation sites.^4–11^

Two mechanisms have been proposed to explain the development of abdominal pregnancies; Intra-abdominal fertilisation with primary implantation of the abdomen (primary abdominal pregnancy) and the more common mechanism of secondary implantation from an aborted tubal pregnancy, termed as secondary abdominal pregnancy.^4,5^ A more useful classification of abdominal pregnancy is based on the gestation age at which it is first detected. Early abdominal pregnancies refer to those first diagnosed at or before 20 weeks while advanced abdominal pregnancies are diagnosed after 20 weeks.^12^

Factors that increase the risk of abdominal pregnancy are similar to those for any ectopic pregnancy and include previous ectopic pregnancy, tubal surgery, history of pelvic inflammatory disease, cigarette smoking, history of infertility and assisted reproductive techniques such as in vitro fertilisation and previous use of an intrauterine device.^13,14^

The clinical presentation of abdominal pregnancy is non-specific, and features vary widely owing to the different possible sites of implantation. Patients may present with nausea, vomiting, vaginal bleeding, abdominal pain, cessation of foetal movements, respiratory problems, haematochezia or may even be asymptomatic. Abdominal pregnancies are usually associated with foetal death, but in very rare cases, abdominal pregnancies result in live births.^14–21^

Diagnostic evaluation of any ectopic pregnancy begins with confirmation of pregnancy in women of reproductive age presenting with vaginal bleeding, abdominal pain, or menstrual irregularities. This is followed by determining the exact location of the extra-uterine pregnancy. Ultrasound is the tool of choice in localizing the pregnancy although the accuracy of diagnosing abdominal pregnancy is as low as 50%, especially in early abdominal pregnancies.^22^ However, Allibone et al^23^ suggest that ultrasonography features that may support the diagnosis of abdominal pregnancy especially in the second trimester include: demonstration of a foetus in a gestational sac outside the uterus, failure to visualize the uterine wall between the foetus and urinary bladder, close proximity between the foetus and the anterior abdominal wall and localisation of the placenta outside the confines of the uterine cavity. Magnetic Resonance Imaging (MRI) may also be helpful in the localisation of abdominal pregnancies. However, its role is limited in low-resource settings.

Localisation of sites of abdominal pregnancies and determining the gestational age at diagnosis play a fundamental role in guiding the treatment approach and minimising potential complications. Surgical management by way of laparotomy remains the mainstay approach to treating second trimester abdominal pregnancies, especially if the foetus is dead as this carries a risk of infection and disseminated intravascular coagulation. The foetus is usually delivered easily, and the key issue lies in the management of the placenta. A recognised approach has been to leave the placenta in situ as attempts to remove it are associated with high risk of fatal haemorrhage. This approach however carries risk of infection and intra-abdominal abscess formation.^21,24^ Postoperative methotrexate may be administered to accelerate involution.^14,21,25,26^

The vague presentation coupled with low accuracy of ultrasound detection often leads to misdiagnosis of abdominal pregnancy,^27–30^ hence highlighting the need for a high index of suspicion and thorough clinical and radiological evaluation to accurately diagnose this condition. We present a case of a second trimester abdominal pregnancy detected following a failed induction for an initial diagnosis of missed abortion, and the ensuing outcome, rarely reported in limited-resource settings.

## CASE PRESENTATION

A19-year-old woman, gravida 2 para 1 with no living children was referred to our institution, a regional referral hospital from a nearby health centre. She presented with loss of foetal movement that began approximately one week prior to admission. Believing this to be a transient state that would return to normal, she did not seek medical care for several days. One day prior to admission, she was attended to at a nearby health centre where an ultrasound was performed. The ultrasound report indicated an intra-uterine pregnancy at gestation age of 17 weeks, with no foetal movements or cardiac activity.

However, an image of the ultrasound was not provided. As the patient was uncertain of her last normal menstrual period, the gestation age determined by ultrasound was adopted. She was subsequently diagnosed to have a missed abortion and referred to our institution for further management.

The cessation of foetal movements was not associated with any other significant symptoms such as abdominal pain or vaginal bleeding. She had not yet booked at antenatal clinic for this pregnancy. Two years ago, she underwent a caesarean section at term. The patient had an intrauterine foetal death but the indication for the caesarean section could not be determined. The underlying cause of the intrauterine foetal death could also not be elucidated. The past medical, surgical, and gynaecological histories were otherwise unremarkable.

General examination findings on admission were essentially normal save for a slightly elevated blood pressure of 130/85mmHg. Per abdominal examination revealed a moderately distended abdomen which was non-uniform, with distension more marked in the suprapubic region. She had a well-healed sub-umbilical midline incision (SUMI) scar. Palpation revealed no tenderness and what was assumed to be a fundal height of 18cm, corresponded with the gestational age by ultrasound. Auscultation by foetoscope did not detect any foetal heart sounds. On sterile per vaginal examination, the cervix was found to be medially positioned, firm, thick and closed.

The patient was provisionally diagnosed to have a missed abortion based on clinical findings and referral ultrasound report. Routine laboratory investigations were ordered. Full blood picture revealed microcytic hypochromic anaemia with haemoglobin level of 7.5g/dL (Table 1). Her blood group was B Rh- (negative). Initial management included oral ferrous sulphate (200mg 12-hourly) to correct anaemia as per national guidelines^31^ and 400 μg misoprostol every 6 hours to manage the missed abortion as per World Health Organization (WHO) guidelines.^32^ Intravenous ceftriaxone (1g 12-hourly for 24hours) and intravenous metronidazole (500mg 8-hourly for 24hours) were given for prophylaxis as per departmental protocols. On day two of admission, the patient reported to experience per vaginal bleeding. Her cervix was 3cm dilated and 60% effaced on sterile per vaginal examination. Oxytocin infusion (5IU in 500ml of Ringer's Lactate at 15 drops per minute) was added to her treatment plan as per departmental protocol.

**TABLE 1:** Full Blood Picture Report on Admission

Parameter	Result	Ref. Range	Units
**White Blood Cell Count (WBC)**	**7.29**	**3.50-9.50**	**10^∧^3/μL**
Neutrophils (Neu%)	69.1	40.0-75.0	%
Lymphocytes (Lym%)	26.1	20.0-50.0	%
Monocytes (Mon%)	3.4	3.0-10.0	%
Eosinophils (Eos%)	1.3	0.4-8.0	%
Basophils (Bas%)	0.1	0.0-1.0	%
Absolute Neutrophil Count (Neu#)	5.04	1.80-6.30	10^∧^3/μL
Absolute Lymphocyte Count (Lym#)	1.90	1.10-3.20	10^∧^3/μL
Absolute Monocyte Count (Mon#)	0.25	0.10-0.60	10^∧^3/μL
Absolute Eosinophil Count (Eos#)	0.09	0.02-0.52	10^∧^3/μL
Absolute Basophil Count (Bas#)	0.01	0.00-0.06	10^∧^3/μL
**Red Blood Cell Count (RBC)**	**4.35**	**3.80-5.10**	**10^∧^6/μL**
Haemoglobin (HGB)	7.5 L	11.5-15.0	g/dL
Haematocrit (HCT)	26.2 L	35.0-45.0	%
Mean Corpuscular Volume (MCV)	60.2 L	82.0-100.0	fL
Mean Corpuscular Hemoglobin (MCH)	17.3 L	27.0-34.0	pg
Mean Corpuscular Hemoglobin Concentration (MCHC)	28.7 L	31.6-35.4	g/dL
Red cell Distribution Width-Coefficient of Variation (RDW-CV)	22.3 H	11.0-16.0	%
Red cell Distribution Width-Standard Deviation (RDW-SD)	48.2	35.0-56.0	fL
**Platelet Count (PLT)**	**321**	**125-350**	**10^∧^3/μL**
Mean Platelet Volume (MPV)	8.5	6.5-12.0	fL
Platelet Distribution Width (PDW)	10.3	9.0-17.0	fL
Plateletcrit (PCT)	0.274	0.108-0.282	%
Platelet-Large Cell Ratio (P-LCR)	17.6	11.0-45.0	%
Platelet-Large Cell Count (P-LCC)	57	30-90	10^∧^9/μL

Upon evaluation on the third day post admission, the patient still had minimal vaginal bleeding and was experiencing lower abdominal pain. There were no changes on vaginal examination, with the cervix still 3cm dilated and 60% effaced. Treatment with antibiotics, misoprostol and oxytocin was continued. Additional investigations were ordered on the fifth day post admission. A repeat haemoglobin level was 6.0g/dL; serum creatinine (2.08mmol/L) and Blood Urea Nitrogen (19.06μmol/L) were within normal limits. An ultrasound was done and reported a non-gravid and bulky uterus having thickened endometrium, free fluid in peritoneal cavity with a non-viable foetus having gestation age of 22 weeks and 5 days. Consequently, the diagnosis was one unit of blood transfusion and was scheduled for a laparotomy which was performed on the seventh day post admission.

**FIGURE 1.**
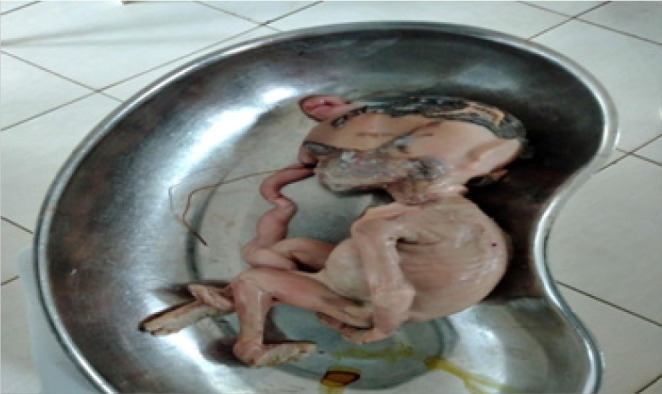
Foetus Extracted During Laparotomy for Omental Pregnancy

Intra-operative findings of the laparotomy included adhesions from the previous Caesarean section for which adhesiolysis was done. The placenta was found to be implanted on the omentum which was located on the anterior abdomen and a macerated foetus was found enclosed in its membranes. The foetus was extracted and weighed 320 grams (Figure 1). The placenta and membranes were left in situ. The abdomen was closed in layers and estimated blood loss was 150 milliliters. The instituted post-operative management included intravenous ceftriaxone (1g 12-hourly), intravenous gentamicin (80mg 24-hourly) and intravenous metronidazole (500mg 8-hourly) for five days for prophylaxis. Intramuscular tramadol (100mg 8-hourly) was prescribed as analgesia for 24 hours and there after she continued with oral analgesics. Oral ferrous sulphate 200mg 8-hourly was administered to correct anaemia. Additionally, she received intravenous 1L Ringer's Lactate interchangeable with Normal Saline for 24 hours, methotrexate (MTX) (50mg/m^2^ intramuscular once daily for three days and then continued with oral methotrexate on day 5, 7, 14, 21 and 28 to reduce vascularity and promote absorption of the retained placenta. She was also transfused with an additional unit of blood.

On the second post-operative day, the patient developed a slight fever of 38.5°C. The surgical site did not have abnormal discharge, and further examination revealed nothing significant except for mild tenderness at the incision site. A repeat full blood picture on the third day post-operation showed normocytic, hypochromic anaemia with haemoglobin level of 9.0g/dL, leucocytosis of 12.33 × 10^∧^^3^/uL (normal laboratory range 3.5-9.5 x 10^∧^^3^/uL) with neutrophilia of 82% (normal laboratory range 40-75%) (Table 2). Her abdomen was slightly distended and tender at the incision site, with no abnormal discharge from the incision site.

**TABLE 2:** Day 3 Post-Operative Full Blood Picture Report

Parameter	Result	Ref. Range	Units
**White Blood Cell Count (WBC)**	**12.33 H**	**3.50-9.50**	10^∧^3/μL
Neutrophils (Neu%)	82.0 H	40.0-75.0	%
Lymphocytes (Lym%)	14.4 L	20.0-50.0	%
Monocytes (Mon%)	3.1	3.0-10.0	%
Eosinophils (Eos%)	0.2 L	0.4-8.0	%
Basophils (Bas%)	0.3	0.0-1.0	%
Absolute Neutrophil Count (Neu#)	10.11 H	1.80-6.30	10^∧^3/μL
Absolute Lymphocyte Count (Lym#)	1.78	1.10-3.20	10^∧^3/μL
Absolute Monocyte Count (Mon#)	0.38	0.10-0.60	10^∧^3/μL
Absolute Eosinophil Count (Eos#)	0.02	0.02-0.52	10^∧^3/μL
Absolute Basophil Count (Bas#)	0.04	0.00-0.06	10^∧^3/μL
**Red Blood Cell Count (RBC)**	**3.01 L**	**3.80-5.10**	**10^∧^6/μL**
Haemoglobin (HGB)	9.0 L	11.5-15.0	g/dL
Hematocrit (HCT)	26.3 L	35.0-45.0	%
Mean Corpuscular Volume (MCV)	87.4	82.0-100.0	fL
Mean Corpuscular Hemoglobin (MCH)	30.0	27.0-34.0	pg
Mean Corpuscular Hemoglobin Concentration (MCHC)	34.3	31.6-35.4	g/dL
Red cell Distribution Width-Coefficient of Variation (RDW-CV)	13.0	11.0-16.0	%
Red cell Distribution Width-Standard Deviation (RDW-SD)	40.8	35.0-56.0	fL
**Platelet Count (PLT)**	**273**	**125-350**	10^∧^3/μL
Mean Platelet Volume (MPV)	10.1	6.5-12.0	fL
Platelet Distribution Width (PDW)	11.6	9.0-17.0	fL
Plateletcrit (PCT)	0.275	0.108-0.282	%
Platelet-Large Cell Ratio (P-LCR)	26.4	11.0-45.0	%
Platelet-Large Cell Count (P-LCC)	72	30-90	10^∧^9/μL

On the fifth post-operative day the patient developed pus-like discharge from the incision site. By this time, she received only a single dose of methotrexate and no additional blood transfusion due to shortages at the facility. The wound was opened up to the subcutaneous layer, and a copious amount of pus was drained, and the wound was cleaned. Pus swab for culture and sensitivity could not be taken due to the patient's financial constraints. An additional diagnosis of septic gapped wound was made. She was prescribed intravenous ciprofloxacin (400mg 12-hourly), metronidazole (500mg 8-hourly) and gentamicin (160mg 24-hourly). She also received one unit of blood transfusion and continued with daily wound dressing. Her condition improved and fourteen days after the initial laparotomy she was scheduled for secondary closure of the wound. On the fifteenth post-operative day, the patient was stable and discharged with oral antibiotics and ferrous sulphate and advised to return for follow-up after two weeks.

## DISCUSSION AND CONCLUSION

Clinically, abdominal pregnancy may be classified based on the gestation age at which it is first detected. Early abdominal pregnancies refer to those first diagnosed at or before 20 weeks while advanced abdominal pregnancies are diagnosed after 20 weeks.^12^ The significance of an accurate gestational age is universally recognised. It aids in formulation of diagnoses, allowing comparison with different literature and determines the timing for interventions including elective deliveries.

Unfortunately, in our case, categorisation of the abdominal pregnancy could not be made as the patient could not recall her last normal menstrual period, and the ultrasound evaluations performed less than a week apart gave a discrepancy of about five weeks, initially 17 weeks and then 22 weeks. This is a clear illustration of a common challenge in obstetrics practice in low-resource settings where dating scans are not routinely performed,^33^ and women present late for first antenatal visits.^34^

In our case the patient presented with a single symptom of cessation of foetal movements. However, different case reports have documented various clinical presentations, including gastrointestinal symptoms, vaginal bleeding, respiratory problems, haematochezia or asymptomatic presentation.^14–21^ This further complicates the diagnostic formulation.

Ultrasound is the imaging investigation of choice in localising the pregnancy although the accuracy of diagnosing abdominal pregnancy is as low as 50%, especially in early abdominal pregnancies.^22^ There have been case reports in our review of the literature that documented a ultrasonography diagnosis of abdominal pregnancy pre-operatively as was found in our case.^14,35^ Although it is not uncommon for the diagnosis to be made intra-operatively both in women who had no prior ultrasound conducted and those with a history of repeated ultrasounds.^14,15,36,37^ This further supports the need for high index of suspicion among clinicians when making a diagnosis of abdominal pregnancy.

While surgical management is the mainstay for treating omental pregnancies, several approaches have been reported, particularly concerning the placenta such as resection with or without partial omentectomy, and leaving the placenta in situ with or without administration of methotrexate.^14,21^ In our case, following extraction of the foetus, the placenta was left in situ and postoperative methotrexate was administered, although not as prescribed due to resource constraints. For the same reason, the patient's anaemia was not sufficiently corrected. This may have contributed to the surgical wound dehiscence and infection.

This case demonstrates the challenges in diagnosing abdominal pregnancies in resource limited settings, and sheds some light on the broader challenges in maternal and perinatal health in developing countries. Delays in initiation of antenatal care is still a prevailing public health concern in our country where only 24% of women start antenatal care before the fourth month of pregnancy and 26% do not seek antenatal care until at least the sixth month of pregnancy.^38^

Several factors contribute to this delay in the decision to seek care, including distance to health facilities, socio-cultural beliefs and lack of awareness about pregnancy complications and not knowing when pregnant women should seek help.^39^ This “Type I delay” according to the “Three delays model” conceptualized by Thaddeus and Maine^40^ plays a significant role in maternal mortality, especially in low-resource settings. The persistence of this and other delays to timely obstetric management calls for enhanced community awareness and sensitisation efforts, especially in hard-to-reach areas.

“Type III delay, the delay in the provision of adequate care^40^ was evident in this case. Although the management of this case was successful in preventing a maternal death, there are a number of challenges that hindered effective and efficient management. These factors, exhibited in this case were shortages of essential drugs and supplies that led to the sub-optimal provision of methotrexate and blood transfusion products. Administrative barriers as exhibited by the almost week-long delay in obtaining an ultrasound and sub-optimal information in patient files e.g., absence of ultrasound images to allow verification of imaging reports; and shortage of qualified and trained personnel that would have enabled early diagnosis and management.

In conclusion, the diagnosis of abdominal pregnancy remains a challenge in resource limited settings and requires a high index of suspicion, especially due to the variability of its presentation. This case emphasizes the need for clinicians to conduct comprehensive assessments of patients, receive capacity building training on obstetric ultrasound skills and continued advocacy for early initiation and provision of quality of antenatal care.
